# Emergency room endotracheal intubation in children with bronchiolitis: A cohort study using a multicenter database

**DOI:** 10.1002/hsr2.169

**Published:** 2020-06-30

**Authors:** Marla R. Carter, Aamer H. Khan, Tarek Salman, Richard Speicher, Alexandre T. Rotta, Steven L. Shein

**Affiliations:** ^1^ Division of Pediatric Critical Care Medicine, Department of Pediatrics Rainbow Babies and Children's Hospital Cleveland Ohio

**Keywords:** bronchiolitis, infections, mechanical ventilation, pediatric critical care, pulmonology

## Abstract

**Background and Aims:**

Bronchiolitis and asthma have a clinical overlap, and it has been shown that pediatric intensive care unit (PICU) patients with asthma undergoing endotracheal intubation in a community hospital emergency room (ER) have a shorter duration of mechanical ventilation (MV) and PICU length of stay (LOS) vs children undergoing intubation in a children's hospital. We aimed to determine if the setting of intubation (community vs children's hospital ER) is associated with the duration of MV and PICU LOS among children with bronchiolitis.

**Methods:**

With IRB approval, data in the Virtual Pediatric Systems (VPS, LLC) database were queried for bronchiolitis patients <24 months of age admitted to one of 103 predominantly North American PICUs between 1/2009 and 1/2016 who had an endotracheal tube in place at PICU admission. There were no exclusion criteria. Extracted data included ER type (community/external or children's hospital/internal), demographics, and reported comorbidities. Outcomes analyzed were duration of MV and PICU LOS. Multivariable linear regression was used to evaluate if intubation location was independently associated with the outcomes of interest.

**Results:**

Among 1934 patients, median age was 2.0 (IQR: 1.0‐4.8) months, 51% were admitted from an external ER, 41% were White, 61% were male, and 28% had ≥1 comorbidity. Median duration of MV was 6.6 (4.6‐9.5) days and the median PICU LOS was 7.0 (4.6‐10.6) days. Children who underwent endotracheal intubation in a children's hospital ER had a modestly longer duration of MV (6.7 [4.4‐9.4] vs 6.5 [5.2‐9.6] days, *P* < .001, Mann‐Whitney *U*) and longer PICU LOS (7.2 [4.8‐10.8] vs 6.9 [4.2‐10.1] days, *P* = .004, Mann‐Whitney *U*). After adjusting for confounding variables, we did not observe a significant association between the location of endotracheal intubation and duration of MV or PICU LOS.

**Conclusion:**

In this cohort, and unlike outcomes of near‐fatal asthma, we observed that clinical outcomes of critical bronchiolitis were similar regardless of location of endotracheal intubation.

## 1 INTRODUCTION

Bronchiolitis is the leading cause of infant hospitalization in the United States and consumes significant health care resources.^1^, ^2^ Among United States children, there are approximately 125 000 to 150 000 admissions for bronchiolitis yearly, with annual costs of over 1.7 billion dollars.^1^, ^2^, ^3^ Children with chronic lung disease, congenital heart disease, prematurity, chronic encephalopathy, and younger age are at an increased risk for severe bronchiolitis, but the majority of cases occur in otherwise healthy children.^4^, ^5^, ^6^, ^7^, ^8^, ^9^, ^10^, ^11^ Approximately 10% of children hospitalized with bronchiolitis require invasive mechanical ventilation (MV), which has been associated with longer pediatric intensive care unit (PICU) and hospital lengths of stay, exposure to possibly neurotoxic sedative medications, and adverse events, like ventilator‐induced lung injury and ventilator‐associated infections.^8^, ^12^, ^13^, ^14^, ^15^, ^16^, ^17^

Recommended therapies for children hospitalized with bronchiolitis are mostly supportive, and practices vary widely.^11^, ^18^, ^19^ There are no interventions that have been proven to reduce the need for MV, but both high‐flow nasal cannula (HFNC) and noninvasive ventilation (NIV) such as continuous positive airway pressure (CPAP) are increasingly used therapies that have been associated with reduced rates of MV compared to historical controls.^20^, ^21^, ^22^ Both HFNC and NIV require equipment and expertise that may not be available for children in all settings, including emergency departments (ED) of community (referral) hospitals and during interfacility transport.

Intubation in a community ED is associated with shorter durations of MV and PICU care compared to intubation in a children's hospital among children with asthma, a condition which similarly can cause wheezing, obstructed airways, and acute respiratory failure.^4^, ^23^ A possible explanation for these findings is that referral ED physicians may have a lower threshold for endotracheal intubation, and therefore, treatment at a children's hospital may enable intubation to be avoided in select cases. As clinical overlap between bronchiolitis and asthma is well recognized,^24^ we utilized a large, multicenter database to determine if location of endotracheal intubation is similarly associated with the duration of MV and PICU length of stay (LOS) in children with bronchiolitis.

## 2 METHODS

This cohort study was reviewed and approved by University Hospitals Cleveland Medical Center Institutional Review Board. Deidentified patient data were obtained with permission from Virtual PICU Systems (VPS, LLC). VPS is an international database that prospectively collects and manages standardized clinical data from over 135 PICUs for the purpose of quality improvement and research.^25^ With the exception of three centers in Saudi Arabia, all contributing centers are located in the United States or Canada. Participation in VPS is voluntary and, therefore, may not be fully representative of all PICUs, but approximately one‐third of all US PICUs participate in VPS and it has supported several prior research studies.^14^, ^15^, ^26^

We reviewed the VPS database for all patients with a primary diagnosis of bronchiolitis between January 2009 and January 2016, as this was the period of data available from VPS when this study was conceived. Patients were included in the analysis if they were 0 to 24 months of age, admitted from an ED to a participating PICU, and had an endotracheal tube in place at the time of PICU admission. There were no exclusion criteria. Children admitted to the PICU from a general ward or any other non‐ED locations were excluded. For each subject, we identified whether they were admitted from the ED of a community hospital or from the ED of a children's hospital. Age, sex, race/ethnicity (as recorded at each VPS center), and comorbidities^15^ were also included in our analysis.^14^ As previously described, the presence of a significant comorbidity was identified using diagnosis codes that were “present at admission.” Severity of illness was evaluated using the Pediatric Index of Mortality‐2 (PIM‐2) score. The primary outcome measure was duration of MV, which was defined as time from endotracheal intubation to time of discontinuation of invasive MV. Extubation was considered successful and MV terminated if the patient did not require reintubation within 48 hours.^27^ The secondary outcome was PICU LOS. VPS defines LOS using the interval between the time of PICU admission and either the time that the patient was deemed medically ready for PICU discharge (“medical LOS”) or the time that the patient physically left the PICU (“physical LOS”). When both measurements were available but differed, medical LOS was used in our analyses.

Differences between children undergoing endotracheal intubation at a community hospital ED and children undergoing endotracheal intubation at a children's hospital ED were identified with Mann‐Whitney *U* test (continuous variables) and chi‐square test (categorical variables). Variables associated with the primary and secondary outcomes were identified in univariate analysis using the Mann‐Whitney *U* test or the chi‐square test, as appropriate. Multivariable linear regression models were created to evaluate the outcomes of interest. A priori, the following variables that are associated with unfavorable courses of bronchiolitis were selected for inclusion in the primary model, using directed acyclic graphs: age, weight, race/ethnicity (as recorded at each VPS center), and PIM‐2 score.^25^, ^28^, ^29^ Secondarily, variables loosely associated (*P* < .10) with the outcome were included in separate multivariable linear regression models. Data are shown as n (%) and median (interquartile range). A *P*‐value of less than .05 was deemed significant. Statistical analysis was performed using SigmaPlot v12.5 (Systat Software Inc., San Jose, California).

## 3 RESULTS

During the study period, we identified 1958 PICU patients with a primary diagnosis of bronchiolitis who were admitted from an ED with an endotracheal tube in place. We excluded 24 patients due to incomplete data, leaving 1934 subjects from 103 centers for analysis. Overall, the median age of the analyzed group was 2.0 (1.0‐4.8) months, 41.1% of patients were White, and 60.9% were male. Approximately one‐quarter (538 [27.5%]) of subjects had at least one identified comorbidity. Among included subjects, 952 patients (49.2%) were admitted from a community hospital ED and 982 (50.8%) were admitted from a children's hospital ED. Children who were intubated in a community hospital ED were significantly older (median: 2.1 [IQR: 1.1‐5.8] vs 1.9 [1.0‐4.2] months, *P* = .003, Mann‐Whitney U) and race/ethnicity differed between the groups (Table 1), but there were no statistically significant differences in prevalence of comorbidities or PIM‐2 score.

**Table 1:** Patient demographics

	Children's Hospital ED N = 982	Community Hospital ED N = 952	*P*‐value
Male	585 (60%)	593 (62%)	.239*
Race/Ethnicity			
White	386 (39%)	410 (43%)	<.001*
African‐American	202 (21%)	93 (10%)	
Hispanic	187 (19%)	151 (16%)	
Other/Unknown	207 (21%)	284 (30%)	
Age (months)	1.9 (1.0‐4.2)	2.1 (1.1‐5.8)	.003**
Comorbidity			
Cardiovascular	70 (7%)	80 (8%)	.336*
Genetic	23 (2%)	12 (1%)	.107*
Immunologic	9 (<1%)	6 (<1%)	.647*
Neurologic	56 (6%)	61 (6%)	.579*
Prematurity	157 (16%)	139 (15%)	.433*
Pulmonology	22 (2%)	28 (3%)	.408*
Any comorbidity	264 (27%)	274 (29%)	.379*
PIM2 probability of death (%)	0.92 (0.73‐1.26)	0.95 (0.75‐1.26)	.592**

*Note*: Data shown as n (%) or median (IQR). As described in Section 2, categorical variables were compared with chi‐square test (*) and continuous variables were compared with Mann‐Whitney *U* test (**).

Abbreviations: ED, emergency department; PIM2, pediatric Index of mortality‐2 score (converted into predicted risk of mortality).

Overall, the median (IQR) duration of MV was 6.6 (4.6‐9.5) days, the median (IQR) PICU LOS was 7.0 (4.6‐10.6) days, and 12 (0.6%) children died prior to PICU discharge. The univariate analyses of variables associated with the duration of MV are shown in Table 2. Children who underwent endotracheal intubation in the children's hospital ED had a modestly longer duration of MV (6.7 [5.2‐9.6] vs 6.5 [4.4‐9.4] days, *P* < .001) compared to children admitted from a community hospital ER. Results of the primary multivariate linear regression model (Table 3) showed that younger age, lower weight, and African‐American race/ethnicity were independently associated with longer duration of MV for children with bronchiolitis. However, we observed that site of endotracheal intubation was not significantly associated with duration of MV after adjusting for significant covariates in the primary model. In the secondary model, while we observed that several comorbidities (genetic, cardiac, and pulmonary) were significantly associated with longer MV duration, location of intubation was not (Table 3).

**Table 2:** Univariate analysis and duration of mechanical ventilation

	Duration of mechanical ventilation (days)	*P*‐value
Children's Hospital ED	6.7 [5.2‐9.6]	<.001**
Community Hospital ED	6.5 [4.4‐9.4]	
Male	6.6 [4.6‐9.4]	.249**
Female	6.6 [4.6‐9.7]	
Race/Ethnicity—White	6.6 [4.6‐9.5]	.097**
African‐American	7.4 [4.6‐10.5]	
Hispanic	6.6 [4.6‐9.5]	
Other/Unknown	6.5 [4.5‐8.7]	
Comorbidity—Cardiovascular—present	8.4 [5.5‐12.8]	<.001**
Absent	6.6 [4.6‐9.4]	
Comorbidity—Genetic—present	9.4 [5.5‐13.4]	.004**
Absent	6.6 [4.6‐9.5]	
Comorbidity—Immunologic—present	7.5 [5.3‐11.6]	.254**
Absent	6.6 [4.6‐9.5]	
Comorbidity—Neurologic—present	6.3 [2.5‐10.5]	.070**
Absent	6.6 [4.6‐9.5]	
Comorbidity—Prematurity—present	7.5 [5.4‐10.5]	.001**
Absent	6.6 [4.6‐9.5]	
Comorbidity—Pulmonary—present	10.4 [6.2‐16.8]	<.001**
Absent	6.5 [4.5‐9.4]	
Comorbidity—Any—present	7.4 [4.6‐10.5]	<.001**
Absent	6.5 [4.5‐9.1]	

*Note*: Data shown as median (IQR). As described in Section 2, categorical variables were compared with chi‐square test (*) and continuous variables were compared with Mann‐Whitney *U* test (**).

Abbreviation: ED, emergency department.

**Table 3:** Multivariable analysis and duration of mechanical ventilation

	Primary model		Secondary model	
	Coefficient	*P*‐value	Coefficient	*P*‐value
Community Hospital ED	−0.373	.145	−0.396	.115
PIM2 score	0.009	.759	0.002	.930
Age	0.123	.016	0.029	.577
Weight (kg)	−0.459	<.001	−0.333	<.001
White	0.268	.399	0.255	.413
African‐American	0.812	.046	0.771	.054
Hispanic	0.586	.136	0.554	.150
Cardiovascular comorbidity			2.166	<.001
Genetic			3.451	<.001
Neurologic			0.888	.100
Prematurity			0.098	.786
Pulmonary			4.525	<.001

*Note*: Multivariable linear regression coefficients greater than zero with a significant *P*‐value (see Section 2) support that the variable is associated with a longer duration of mechanical ventilation. Variables were selected a priori, using directed acyclic graphs, for the primary model, and using the results of the univariate analyses for the secondary model.

Abbreviations: ED, emergency department; PIM2, pediatric index of mortality‐2 score.

Variables associated with PICU LOS in univariate analyses are shown in Table 4. Children undergoing endotracheal intubation in a children's hospital ED had a longer PICU LOS (7.2 [4.8‐10.8] vs 6.9 [4.2‐10.1] days, *P* = .004) compared to those intubated in a community hospital ED. PICU LOS was analyzed using the “medical LOS” definition in 1858 cases (96%) vs “physical LOS” in 76 cases (4%). In both the primary and secondary multivariable linear regression models (Table 5), we observed that the site of endotracheal intubation was not significantly associated with PICU LOS after adjusting for a priori defined and significant covariates, respectively.

**Table 4:** Univariate analysis and PICU LOS

	PICU LOS (days)	*P*‐value
Children's Hospital ED	7.2 [4.8‐11.0]	.004**
Community Hospital ED	6.8 [4.2‐10.1]	
Male	6.9 [4.5‐10.0]	.166**
Female	7.3 [4.7‐10.9]	
Race/Ethnicity—White	6.9 [4.6‐10.7]	.121**
African‐American	7.7 [4.6‐10.7]	
Hispanic	6.8 [4.4‐9.8]	
Other/Unknown	6.9 [4.6‐9.9]	
Comorbidity—Cardiovascular—present	9.7 [5.8‐15.0]	<.001**
Absent	6.9 [4.5‐10.0]	
Comorbidity—Genetic—present	11.8 [7.5‐16.2]	<.001**
Absent	7.0 [4.6‐10.3]	
Comorbidity—Immunologic—present	12.5 [7.0‐15.1]	.009**
Absent	7.0 [4.6‐10.6]	
Comorbidity—Neurologic—present	6.7 [3.3‐14.0]	.983**
Absent	7.0 [4.6‐10.1]	
Comorbidity—Prematurity—present	7.5 [5.4‐11.5]	.002**
Absent	6.9 [4.5‐10.2]	
Comorbidity—Pulmonary—present	6.9 [4.6‐10.2]	<.001**
Absent	8.1 [7.3‐20.1]	
Comorbidity—Any—present	8.1 [5.1‐12.6]	<.001**
Absent	6.8 [4.5‐9.6]	

*Note*: Data shown as median (IQR). As described in Section 2, categorical variables were compared with chi‐square test (*) and continuous variables were compared with Mann‐Whitney U test (**).

Abbreviations: ED, emergency department; LOS, length of stay; PICU, pediatric intensive care unit.

**Table 5:** Multivariable analysis and PICU LOS

	Primary model		Secondary model	
	Coefficient	*P*‐value	Coefficient	*P*‐value
Community Hospital ED	0.316	.335	−0.384	.208
PIM2 score	0.037	.298	0.036	.293
Age	0.194	.002	0.095	.132
Weight (kg)	−0.593	<.001	−0.450	<.001
Cardiovascular comorbidity			2.933	<.001
Genetic			4.654	<.001
Immunologic			2.485	.153
Prematurity			−0.183	.681
Pulmonary			6.012	<.001

*Note*: Multivariable linear regression coefficients greater than zero with a significant *P*‐value (see Section 2) support that the variable is associated with a longer PICU length of stay. Variables were selected a priori, using directed acyclic graphs, for the primary model, and using the results of the univariate analyses for the secondary model.

Abbreviations: ED, emergency department; LOS, length of stay; PICU, pediatric intensive care unit; PIM2, pediatric index of mortality‐2 score.

## 4 DISCUSSION

We used a large, multicenter PICU database to study children with bronchiolitis undergoing endotracheal intubation before PICU admission and found that intubation in a community ED was associated with a statistically significant increase in duration of MV and PICU length of stay. However, these differences of a few hours are unlikely to be clinically relevant, and the associations were not statistically significant after adjusting for covariates such as age, race, comorbidities, and PIM2 score. This was true in both models including variables selected a priori using directed acyclic graphs and secondary models where variables were selected based on univariate analyses, including comorbidities.^28^

Several factors may influence the decision to initiate mechanical ventilation in a child with acute respiratory distress from diseases like bronchiolitis, and all of these factors may play a role in our findings. It is intuitive that clinicians with less pediatric‐specific training may have a different threshold to perform endotracheal intubation due to different perceptions about severity of illness and the risk of decompensation. Performing endotracheal intubation prior to interhospital transfer may be seen as a way to increase patient safety.^30^, ^31^ Potential lack of equipment, reduced pediatric‐specific training, and optimizing safe transport may lead to an association between endotracheal intubation at a community hospital ED and faster liberation from MV, as they may all lead to less ill children undergoing endotracheal intubation.

We did not observe such an association between endotracheal intubation at a community hospital ED and faster liberation from MV, which differs from prior studies of children with asthma.^4^, ^23^ In a single center study, Carroll et al found that children with near‐fatal asthma intubated at a community hospital ED had a shorter duration of MV and PICU LOS.^4^ Similar findings were reported in a subsequent study that used the VPS database.^23^ These authors suggested that their findings may relate to differences in the threshold to perform endotracheal intubation. However, our data do not support the existence of a significant difference in thresholds, at least among children with bronchiolitis. One might have expected this difference to be more profound in our cohort, given that infants and young children with bronchiolitis may be even less like “little adults” than older children with near‐fatal asthma because bronchiolitis patients are even younger and have even more different physiology.^32^ Future studies in children with different diseases (eg, pneumonia, status epilepticus, septic shock) could provide additional evidence to support or refute differences in thresholds for endotracheal intubation in various settings.

It is possible that our findings may relate to differences in ventilator weaning. Prompt extubation of some children with near‐fatal asthma is an established practice, but clinicians may not be similarly inclined to pursue early liberation in children with bronchiolitis.^33^ Without a clinician‐driven goal of early extubation, the duration of MV in observational studies such as this may not correlate as well with severity of illness. Only a prospective study with protocolized ventilator weaning and mandated assessments of extubation readiness can optimally use duration of MV as a surrogate for severity of lung disease, though such studies are inherently more challenging to complete. Future observational studies may consider using physiologic variables related to MV (eg, oxygenation index, static compliance) as an outcome or to adjust for duration of MV.

Recent studies suggest that clinical outcomes like LOS may be comparable in a cohort of similarly ill children with bronchiolitis, regardless of the type of respiratory support provided. Children with moderate bronchiolitis randomized to receive standard low‐flow nasal cannula therapy vs HFNC had no differences in duration of oxygen therapy or hospital LOS.^34^ Among more severely ill children admitted to the PICU with bronchiolitis, randomization to HFNC vs CPAP was not associated with differences in PICU LOS or duration of ventilation.^35^ Recent observational data supports that PICU LOS is similar between centers even when rates of MV usage differ widely.^36^ These all suggest that a child's general level of illness may be a stronger predictor of LOS than the respiratory support provided.

Strengths of our study include the large size of the cohort and the use of an established, multicenter data source. The generalizability of our findings is supported by the fact that our methods identified risk factors for unfavorable outcomes, including prematurity and other pre‐existing comorbidities, also found in several other studies of patients with bronchiolitis.^6^, ^7^, ^16^, ^37^, ^38^ There are, however, a number of important limitations, most of which are inherent to its retrospective nature.^39^ First, errors in data entry may impact the results of any database study. VPS data, however, are entered by trained personnel and undergo several quality control procedures before inclusion in the database, enabling it to support several prior PICU studies.^14^, ^15^, ^25^ Second, the database does not contain data that the clinician may have considered when deciding to perform endotracheal intubation, such as vital signs, physical exam findings, hypercarbia, presence of apnea, and others, making it prone to confounding by indication. Third, endotracheal intubation and weaning from MV were not protocolized, and prior studies show there is tremendous practice variation in severe bronchiolitis.^40^, ^41^ Fourth, children who underwent endotracheal intubation at a community ED but were transferred to the children's hospital ED and only then to the PICU would have been misclassified in our analysis, though this practice is not widely reported and not available in the VPS database. Fifth, we did not include co‐morbid conditions in the variables selected a priori for our primary multivariable model, since parents of children with such comorbidities may preferentially bring their child to a children's hospital emergency room.^42^ However, the effect of selected comorbidities was evaluated in the secondary linear regression models, and our key finding—that location of endotracheal intubation appeared no to be associated with clinical outcomes—was consistent among all models. Sixth, the VPS database may not be representative of all PICU patients, given, for instance, that participation in VPS is voluntary, though it has supported numerous prior studies of critically ill children.^43^, ^44^, ^45^

## 5 CONCLUSION

In a large, multicenter database of children with critical bronchiolitis undergoing endotracheal intubation before PICU admission, we did not observe that intubation in a community ED was associated with a significant difference in duration of MV or PICU LOS vs intubation in a children's hospital ED. This does not support the presence of differing thresholds for endotracheal intubation between ED providers in various hospital types as previously described among children with asthma. Further studies in other diseases could enable better interpretation of the conflicting findings in children with asthma and help identify factors that may identify children in whom endotracheal intubation may be avoidable, including initiation of HFNC or NIV prior to intrafacility transport.

## CONFLICT OF INTEREST

Dr Alexandre T. Rotta receives personal fees from Vapotherm, Inc., Breas USA, and Elsevier outside of the submitted work. Drs Marla R. Carter, Aamer H. Khan, Tarek Salman, Richard Speicher, and Steven L. Shein disclose that they do not have any potential conflicts of interest. These sources played no role in study design; collection, analysis, and interpretation of data; writing of the report; or the decision to submit the report for publication.

## AUTHOR CONTRIBUTIONS

Conceptualization: Tarek Salman, Richard Speicher, Alexandre T. Rotta, Steven L. Shein

Data curation: Tarek Salman, Steven L. Shein

Formal analysis: Marla R. Carter, Steven L. Shein

Methodology: Tarek Salman, Richard Speicher, Alexandre T. Rotta, Steven L. Shein

Project administration: Steven L. Shein

Supervision: Steven L. Shein

Validation: Richard Speicher, Alexandre T. Rotta, Steven L. Shein

Writing – original draft preparation: Aamer H. Khan, Marla R. Carter, Steven L. Shein

Writing – review and editing: Marla R. Carter, Aamer H. Khan, Tarek Salman, Richard Speicher, Alexandre T. Rotta, Steven L. Shein

All authors have read and approved the final version of the manuscript.

Marla R. Carter had full access to all of the data in this study and takes complete responsibility for the integrity of the data and the accuracy of the data analysis.

## TRANSPARANCY STATEMENT

The lead author (Dr Marla R. Carter) affirms that this manuscript is an honest, accurate, and transparent account of the study being reported; that no important aspects of the study have been omitted; and that any discrepancies from the study as planned have been explained.

## Data Availability

The data that support the findings of this study are available from Virtual PICU Systems (VPS, LLC). Restrictions apply to the availability of these data, which were used under license for this study. Data are available with the permission of VPS. Instructions on how to obtain a license can be obtain from https://www.myvps.org/
